# Prognostic Factors Affecting Mortality Among Patients Admitted to the Intensive Care Unit with Acute Hypoxemic Respiratory Failure

**DOI:** 10.3390/diagnostics15141784

**Published:** 2025-07-15

**Authors:** Kerem Ensarioğlu, Melek Doğancı, Mustafa Özgür Cırık, Mesher Ensarioğlu, Erbil Tüksal, Münire Babayiğit, Seray Hazer

**Affiliations:** 1 Department of Pulmonary Medicine, Ankara Atatürk Sanatoryum Training and Research Hospital, Ankara 06290, Turkey; 2 Department of Anesthesiology and Reanimation, Ankara Atatürk Sanatoryum Training and Research Hospital, Ankara 06290, Turkey; melekdidik@hotmail.com (M.D.); dr.ozgurr@hotmail.com (M.Ö.C.); meshercapras@gmail.com (M.E.); drerbilturksal@hotmail.com (E.T.); dr.mbabayigit@gmail.com (M.B.); 3 Department of Thoracic Surgery, Ankara Atatürk Sanatoryum Training and Research Hospital, Ankara 06290, Turkey; drserayhazer@gmail.com

**Keywords:** acute respiratory failure, APACHE, intensive care unit, mortality, nutritional status

## Abstract

**Background/Objectives:** Acute hypoxemic respiratory failure is a significant condition commonly seen in intensive care units (ICUs), yet specific prognostic markers related to it for mortality remain largely unstudied. This study aimed to identify parameters that influence mortality in ICU patients diagnosed with type 1 respiratory failure. **Methods:** A retrospective cohort study was conducted at a tertiary care hospital, including patients admitted to the ICU between March 2016 and March 2020. The study included patients with type 1 respiratory failure, while exclusion criteria were prior long-term respiratory support, type 2 respiratory failure, and early mortality (<24 h). Data on demographics, comorbidities, support requirements, laboratory values, and ICU scoring systems (APACHE II, SOFA, SAPS II, NUTRIC) were collected. Binomial regression analysis was used to determine independent predictors of 30-day mortality. **Results:** Out of 657 patients screened, 253 met the inclusion criteria (mean age 70.6 ± 15.6 years; 65.6% male). Non-survivors (*n* = 131) had significantly higher CCI scores; greater vasopressor requirements; and elevated SAPS II, APACHE, SOFA, and NUTRIC scores. Laboratory findings indicated higher inflammatory markers and lower nutritional markers (albumin and prealbumin, respectively) among non-survivors. In the regression model, SAPS II (OR: 13.38, *p* = 0.003), the need for inotropic support (OR: 1.11, *p* = 0.048), NUTRIC score (OR: 2.75, *p* = 0.014), and serum albumin (inverse; OR: 1.52, *p* = 0.001) were independently associated with mortality. The model had an AUC of 0.926 and classified 83.2% of cases correctly. When combined, SAPS II and mNUTRIC had more AUC compared to either standalone scoring. **Conclusions:** SAPS II, vasopressor requirements, mNUTRIC score, and low serum albumin are independent predictors of 30-day mortality in patients with acute hypoxemic respiratory failure. These findings support the integration of nutritional assessment, a combination of available scoring systems and comprehensive scoring into routine ICU evaluations for this patient group.

## 1. Introduction

Respiratory failure is often evaluated according to the presence of hypoxia or hypercapnia, with a myriad of possible causes and underlying comorbidities that may contribute to either respiratory failure type [1]. Type 1 respiratory failure occurs when the respiratory system provides inadequate oxygen, which may be caused by alveolar hypoventilation, diffusion defect, ventilation-perfusion mismatch, or right-to-left shunt [2,3]. It is defined by the presence of arterial partial oxygen pressure below 60 mmHg. Hypoxemia and hypoxia are commonly used terms in the presence of type 1 respiratory failure and are often used interchangeably; however, they are not synonymous [2]. Hypoxemia is defined as a condition where arterial partial oxygen pressure is below 80 mmHg, which differs from the definition of type 1 respiratory failure, while hypoxia is defined as failure of oxygenation at the tissue level and cannot be measured directly [1]. Acute hypoxemic respiratory failure, thus, is defined under the first category and requires evaluation by arterial blood gas (ABG) analysis [4]. It differs from hypoxemia in its nature, as respiratory failure is defined by inadequate oxygen delivery that may arise from a myriad of causes; therefore, it may occur despite adequate oxygen support, such as in acute respiratory distress syndrome (ARDS). ARDS itself may be defined as a subtype of acute type 1 respiratory failure with specific recently proposed diagnostic criteria available regarding the evaluation of oxygenation response, additional imaging modalities to be utilized (including ultrasonography), and suggestions for resource-limited settings [5]. As such, the definition of respiratory failures, regardless of clinical outcome, plays an important role in patient management. 

The diagnosis of type 2 respiratory failure, which is described as the inability of the respiratory system to remove carbon dioxide from the system, requires ABG sampling [4]. Type 2 respiratory failure is commonly categorized under two possible pathways: pump failure or increased production, with ventilation insufficiency being another yet commonly encountered cause [3]. It is also categorized similarly to type 1 respiratory failure in terms of acute or chronic failure. Acute hypercapnic respiratory failure is defined by a partial carbon dioxide pressure above 45 mmHg and a pH less than 7.35 in ABG sampling [2].

Both types of respiratory failure are significant in managing patients in the intensive care unit (ICU) [6]. Since respiratory failure is not a disease but an outcome of underlying pathologies that cause insufficient ventilation, prioritizing the control of these underlying conditions is essential [7]. Supporting ventilation during this process is a necessary part of ICU care. Oxygenation and respiratory support through mechanical ventilation (MV) or non-invasive ventilation (NIMV) are mainstream approaches for managing respiratory failure [8]. Most ICU scoring systems include at least one method for evaluating either respiratory failure or the need for support.

Type 2 respiratory failure has been extensively researched, with numerous studies evaluating it either in isolation or alongside commonly encountered comorbidities like chronic obstructive pulmonary disease (COPD) and interstitial lung disease [9,10,11,12,13].

The APACHE II, Sequential Organ Failure Assessment (SOFA), and Simplified Acute Physiology Score (SAPS) II are widely used scoring systems that include parameters related to respiratory failure [14,15,16]. In contrast, other scoring systems, such as modified Nutrition Risk in the Critically Ill (mNUTRIC), may not incorporate parameters [17]. However, specific parameters or scoring systems for patients with type 1 respiratory failure remain limited and are a topic that warrants further exploration. A separate evaluation of patients with hypoxemic respiratory failure may be warranted, especially considering its varying causes that might affect evaluation parameters, which may not be evident on patients without respiratory failure.

Scoring systems for issues in low-resource or ICU settings are especially important, including for acute hypoxemic respiratory failure. The literature includes studies showing that, under optimal management and resource allocation, care for patients in limited settings can improve, even in cases like COVID-19 where medical equipment had to be rationed [18].

This study aims to investigate prognostic factors among patients admitted to the ICU with acute hypoxemic respiratory failure. The research hypothesizes that distinct factors, separate from those routinely examined and utilized in the ICU scoring system parameters, may be significant in evaluating patients with type 1 respiratory failure.

## 2. Materials and Methods

The study was performed as a retrospective cohort in a tertiary training and research hospital after the approval of local ethics committee (approval date 14 May 2025, approval no: 2024-BÇEK/276). To exclude patients diagnosed with Coronavirus Disease 2019 (COVID-19), as the hospital served as a center for the control of patients with COVID-19 and respiratory comorbidities, a four-year period from March 2016 to March 2020 was evaluated. Patients admitted to the hospital’s multiple ICUs formed the study’s population.

The hospital’s multiple ICUs included two units under the anesthesia and reanimation department, with one additional unit coordinated by both the anesthesia and pulmonary medicine departments. All three units cared for the general patient population, with priority given to the interdisciplinary-coordinated ICU when patients with known respiratory conditions needed admission. Along with attending physicians, resident doctors from various specialties were responsible for patient care. Patients stable enough for discharge from the ICU either transferred to a ward for evaluation before hospital discharge under the relevant specialty or, in cases of respiratory failure or known respiratory comorbidities, had the option to be moved to another ICU specialized in pulmonary medicine and respiratory failure, staffed entirely by pulmonary medicine specialists.

The inclusion criteria were being over 18 years and the presence of type 1 respiratory failure upon admission. Patients with known respiratory failure history and being treated for it (long-term oxygen treatment or any types of ventilation support), being admitted with type 2 respiratory failure, having invasive ventilation support requirements, and having inadequate data for scoring system evaluation (patients readmitted from other wards, exitus within 24 h of admission) were excluded from the study. Patients’ demographic information; comorbidities; hospitalization history; duration in days; additional support requirements in ICU; scoring systems, including APACHE II, SOFA, SAPS II, and mNUTRIC; admission laboratory values; and 30-day mortality were recorded from the hospital system and ICU admission records.

### Statistical Analysis

The study hypothesized that there would be factors affecting 30-day mortality among patients with type 1 respiratory failure. Regarding the required sample size for the study to demonstrate a statistical difference between two groups regarding 30-day mortality, with an effect size of 0.4, a 5% type 1 error, and 80% power, at least 78 patients in both survivor and exitus (non-survivor) groups were required. To conduct a binomial regression analysis on mortality, with the same error and power, and assuming an odds ratio of 1.5, at least 250 patients were required, assuming five predictor variables and 20 events per predictor variable.

After patients’ data were evaluated in Microsoft Office 365 Excel for any mis-inputs, IBM SPSS Edition 30 was used as the statistical package. Q-Q plots were utilized for the parametric distribution investigation. Data with a parametric distribution was presented with mean and standard deviation, while non-parametric results were given with median, 25th, and 75th percentiles. A comparison of scale parameters was performed by independent t-tests and Mann–Whitney U according to the distribution pattern. Comparisons between categorical parameters were made by the chi-squared test. Parameters that were observed to be statistically significant in the analysis were later evaluated in binomial regression analysis to investigate possible independent factors regarding mortality. *p* values of less than 0.05 were accepted as statistically significant.

Due to the study design, loss to follow-up was not expected, as the exclusion criteria would remove patients who could not be evaluated, specifically regarding possible limitations of scoring systems due to exitus within the day of admission.

## 3. Results

**Figure 1:**
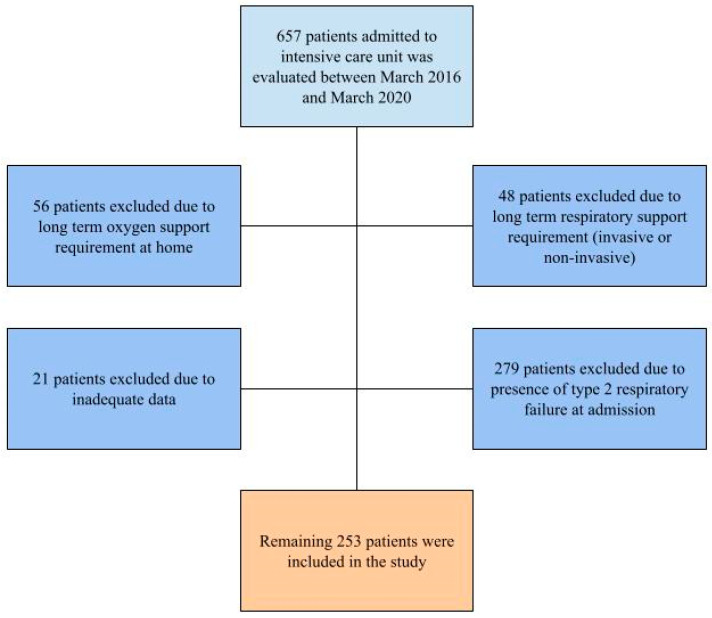
Patient evaluation flow chart.

A total of 657 patients were evaluated in the study. Forty-eight patients were excluded from the study due to long-term respiratory support under non-invasive or invasive mechanical ventilation before admission. An additional 56 patients were removed due to long-term oxygen support requirements at home due to respiratory failure. Twenty-one patients had to be excluded due to either inadequate data and/or exitus before any scoring modality could be performed. Two hundred seventy-nine patients were excluded due to the presence of type 2 respiratory failure (Figure 1).

The remaining 253 patients were included in the study, with 122 survivors and 131 non-survivors observed at the end of the first month. The mean age of the patients was 70.59 (±15.61) years, with the mean age being higher in the non-survivor group, 75 (±11.9) years to 65.85 (±17.66) years (*p* value of 0.001). The group was predominantly male, with 166 (65.64%) male patients to 87 (34.36%) female patients, and there was no difference regarding mortality between sexes. Charlson Comorbidity Index (CCI) score was higher in the non-survivor group (a mean of score 7.57 to 5.12, *p* value of 0.001). Ionotropic and vasopressor requirements were observed rarely in the survivor group (6.5%, *n* = 8) compared to the non-survivor group (55.72%, *n* = 73). Mechanical ventilation support during the ICU stay was over 80% in both groups, with survivors having a higher requirement (95.9% to 82.44%, with a *p* value of 0.001) (Table 1).

Regarding admission duration, survivors had a longer median admission duration in the ward (9 days to 3 days) and total admission duration (13 days to 9 days) compared to the non-survivors, while ICU admission was longer in non-survivors, with a median of 4 to 2 days. ICU readmission was similar in both groups (7.1%, 18 patients in total). All scoring systems were found to be higher in the non-survivor group, with mean scores of APACHE II, SOFA, SAPS II, and mNUTRIC being 19.56 (±5.71) to 27.59 (±8.25), 5.56 (±1.19) to 8.72 (±3.07), 42.92 (±11.94) to 60.56 (±12.86), and 4.53 (±1.72) to 6.79 (±1.52), respectively (*p* values of 0.001 for all comparisons performed) (Table 1).

The admission laboratory values had shown a statistical difference between lymphocyte (1.17 × 10^9^/L to 0.7 × 10^9^/L, *p* value: 0.001) and platelet count (279 × 10^9^/L to 197 × 10^9^/L, *p* value: 0.001) between survivor and non-survivor groups. Regarding liver function tests, aspartate transaminase (35 IU/L to 24 IU/L), alanine aminotransferase (20 IU/L to 18 IU/L), total (0.75 mg/dL to 0.6 mg/dL), and direct bilirubin (0.3 mg/dL to 0.2 mg/dL) levels were higher in the non-survivor group (*p* values of 0.001, 0.005, 0.001, and 0.001, respectively). Renal function was observed to be preserved in the survivor group, with lower creatinine (0.8 mg/dL to 1.1 mg/dL) and blood urea nitrogen (19 mg/dL to 42 mg/dL), and higher glomerular filtration rate (83 mL/min/1.73 m^2^ to 60 mL/min/1.73 m^2^), compared to the non-survivor group (*p* value of 0.001 for all comparisons). Inflammatory markers, C-reactive protein (101 mg/dL to 66.5 mg/dL), and procalcitonin (0.85 ng/mL to 0.24 ng/mL) were elevated among the non-survivors compared to the survivors, while albumin (2.64 g/dL to 3.22 g/dL) and prealbumin (7 mg/dL to 14 mg/dL) were observed to be higher among survivors (a *p* value equal to 0.001 for all comparisons) (Table 2).

**Figure 2:**
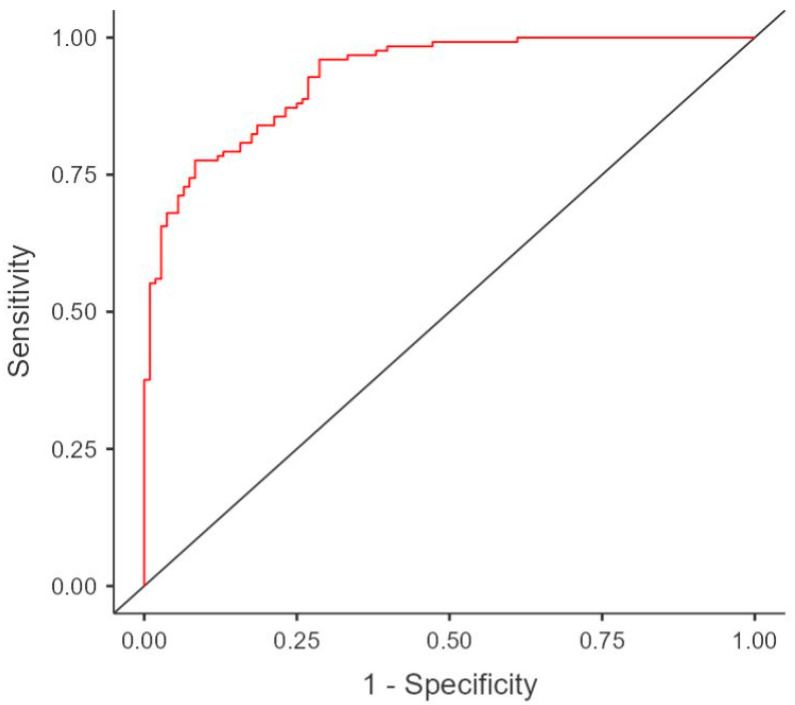
Regression analysis ROC curve.

APACHE II, SOFA, and age were excluded from the regression analysis, as they were already incorporated into the mNUTRIC score, and multicollinearity was present in the model created. The age when accepted also had increased overall variance inflation factors (VIFs) for all parameters; thus, excluding it as a separate parameter while keeping the mNUTRIC score was accepted as a valid statistical approach. Blood urea nitrogen was also excluded, as it is part of SAPS II. The binomial regression model for 30-day mortality included the ionotropic and mechanical ventilation requirements, lymphocyte count, platelet count, total bilirubin, creatinine, SAPS II, CCI, and mNUTRIC score. The model was statistically significant, with a *p*-value of 0.001, X^2^ = 173, and a Nagelkerke’s R^2^ of 0.672. The model had correctly predicted 83.2% of all cases, with a specificity of 81.9% and sensitivity of 83.2%, and had an area under the curve (AUC) of 0.926 (Figure 2).

In the model SAPS II, ionotropic support, mNUTRIC score, and albumin were observed to be independent predictor parameters for 30-day mortality (*p* values of 0.003, 0.048, 0.014, and 0.001; and odds ratio of 13.38, 1.11, 2.75, and 1.52, respectively) (Table 3).

**Figure 3:**
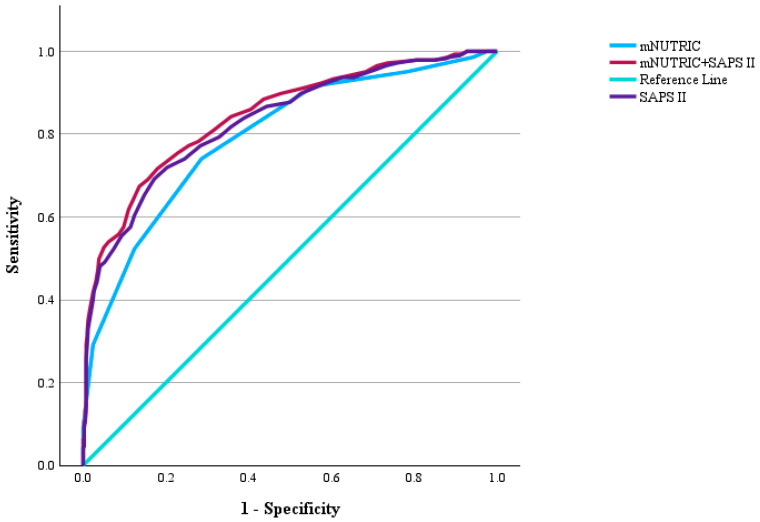
ROC Curve of mNUTRIC, SAPS II, and mNUTRIC + SAPS II.

SAPS II and mNUTRIC scores, when evaluated separately, had a statistically significant AUC for 30-day mortality, with AUC ranging from 0.8 to 0.862 and 0.762 to 0.828, respectively (*p* value of 0.001). Combined, the overall AUC increased to a range of 0.815 to 0.874, remaining statistically significant (*p* value of 0.001). A comparison between SAPS II, mNUTRIC, and the combined scoring value was then performed, and AUC was compared between pairs using Delong analysis. There was no statistically significant difference between SAPS II and mNUTRIC; however, the combined score had a higher AUC compared to the two standalone scoring systems (Table 4 and Figure 3).

## 4. Discussion

In the conducted regression analysis, SAPS II, ionotropic support, mNUTRIC score, and albumin were identified as independent risk factors for 30-day mortality among patients with acute hypoxemic respiratory failure. While we believe that, as indicated in the previous analysis, APACHE II and SOFA would also contribute to the evaluation, a regression model for these scoring systems could not be developed to support this assertion.

The literature, as stated in the introduction, remains limited in evaluating type 1 or acute hypoxemic respiratory failure mortality. However, studies are prevalent regarding the comparison of both types of respiratory failure and examining them in between, such as the study by Kim et al., which evaluated patients with COPD requiring admission and having either type 1 or type 2 respiratory failure [19]. The study stated that patients with type 1 had higher mortality and readmission rates compared to type 2 while exhibiting a similar long-term prognosis. Lai et al. reported similar results without dividing respiratory failure types and noted that an APACHE II score over 25, unstable hemodynamic status (therefore requiring IV and ionotropic support), as well as kidney and hepatic failure were considered risk factors for overall in-hospital mortality [6]. An older but high-inclusion study conducted in Sweden, which evaluated all types of respiratory failure, acute lung injury, and ARDS, also supported APACHE II as a mortality evaluation parameter for patients in the ICU setting [20]. Regarding APACHE II, our study supports the available literature; however, as is stated in the limitation section, a regression analysis could not be performed due to utilized parameters for evaluation of APACHE II as an independent factor.

SAPS II, unlike APACHE II, has limited studies regarding its utilization in type 1 respiratory failure evaluation, and the available literature varies regarding its role. Studies examining its implications in the clinical context were conducted for veno-arterial extracorporeal oxygenation by Lee et al., and the failure of NIMV among critically ill patients by Menga et al. [21,22]. Both studies supported the notion that SAPS II is an important independent risk factor for overall mortality among patients in the related ICU setting.

Comparison of SAPS II to other scoring systems was also performed, in which Jegal et al. stated similar performance regarding the evaluation of clinical severity among ARDS when SAPS II was compared to APACHE II and the lung injury score [23]. Our study reports a similar result and states that SAPS II, as an independent scoring system, is a reliable and valid method of evaluating patients in acute hypoxemic respiratory failure. As stated earlier, a direct comparison to APACHE II could not be made; however, as supported by the available literature, we believe both scoring systems have merit, with SAPS II being more statistically supported in our study. The combination of SAPS II and mNUTRIC being superior over either standalone evaluation in our study also supports possible combinations and justifies a similar approach in patient assessment.

Ionotropic support, regardless of the underlying cause, has always been considered a risk factor for ICU and overall mortality in the literature [24,25]. The observation of this in our study was not viewed as an atypical finding and aligns with current clinical knowledge. Nutritional support, on the other hand, has garnered considerable interest, with many recent studies highlighting its importance and contributing to previously established knowledge in general care [26,27,28]. The need for such support is clear, as various guidelines and studies endorse both adequate support and early assessment methods for determining the need for assistance. In addition to routinely ordered blood tests, which often include albumin and/or prealbumin, scoring systems like mNUTRIC have been utilized in ICU mortality prediction [29,30]. While studies exist examining overall mortality through mNUTRIC in ICU settings, specific research has also focused on respiratory failure. Kumar et al. discussed the role of mNUTRIC in patients with ARDS related to Coronavirus Disease 2019 (COVID-19), and similarly, Todur et al. reported a modified version of mNUTRIC as an independent predictor for patients with ARDS [31,32]. Investigations into the role of mNUTRIC in acute hypoxemic respiratory failure as a distinct entity remain limited, and our research supports the use of mNUTRIC as an independent assessment tool for mortality in this patient group.

Regarding the step-down unit and care provided after ICU, we believe that patients received adequate support after ICU care, with relevant specialties attending to them before hospital discharge. Due to the nature of our tertiary center, further specialized care was provided for patients with respiratory failure in a medical ICU, which may not be possible in other low-resource settings.

Overall, the main limitations of the study include it being conducted at a single center, its retrospective design, and the absence of additional data that could have influenced patient outcomes. Another limitation was excluding APACHE II and SOFA scores from the regression model. These scores could not be shown as separate and independent risk factors when other parameters were included, mainly due to overlapping variables in other scoring systems and high multicollinearity. We were unable to develop a separate model that included only nutritional status and these scoring systems because its reliability and predictive power were considered too low. We believe this issue would remain regardless of study size because of the statistical evaluation method used. Nonetheless, these scoring systems are well-established and reliable, having been effectively used in ICU settings. Therefore, although they may not function as independent risk factors, they still significantly contribute to assessing patients with type 1 respiratory failure. The absence of other potentially relevant parameters, such as CCRT, the exact cause of AHRF, the P/F ratio, and respiratory system compliance, which could influence overall mortality in both the general population and those admitted to the ICU with respiratory issues, also constitutes a limitation of the study.

## 5. Conclusions

In patients with acute hypoxemic respiratory failure in the ICU, SAPS II score, vasopressor requirements, mNUTRIC score, and serum albumin levels were identified as independent predictors of 30-day mortality. Although other ICU scores, such as APACHE II and SOFA, were found to be higher in non-survivors, their overlap with other variables limited their effectiveness as independent predictors in regression analysis. These results emphasize the importance of considering nutritional status and organ support requirements early in the management of patients with type 1 respiratory failure. Care given after ICU, especially in step-down units, may also contribute to the availability and utilization of evaluation scoring systems. Additionally, the study advocates for the incorporation of comprehensive scoring systems and nutritional assessments in prognostic evaluations, with possible combinations of scoring systems being justifiable.

## Figures and Tables

**Table 1:** Demographic parameters, support requirements, admission evaluation, and scoring systems.

Parameters (*n*, SD)		Survivors (*n* = 122)	Non-Survivors (*n* = 131)	Total (*n* = 253)	*p* Value
Demographics					
Gender (%)	Female	46 (37.7)	41 (31.29)	87 (34.36)	0.284
	Male	76 (62.3)	90 (68.71)	166 (65.64)	
Age (Years)		65.85 (17.66)	75 (11.9)	70.59 (15.61)	0.001
Charlson Comorbidity Index Score		5.12 (1.86)	7.57 (2.65)	6.39 (2.6)	0.001
Additional Support Requirements					
Inotropes and Vasopressors		8 (6.55)	73 (55.72)	81 (32)	0.001
Mechanical Ventilation		117 (95.9)	108 (82.44)	225 (88.93)	0.001
Admission Duration (Days)					
Intensive Care Unit		2 (1–5)	4 (1–7)	3 (1–6)	0.01
Ward		9 (6–16)	3 (0–11)	7 (1–15)	0.001
Total Admission		13 (9–22)	9 (4–22)	12 (5.5–22)	0.015
ICU Readmission (%)		10 (8.19)	8 (6.1)	18 (7.1)	0.54
Scoring Systems					
APACHE II		19.56 (5.71)	27.59 (8.25)	23.72 (8.18)	0.001
SOFA		5.56 (1.19)	8.72 (3.07)	7.19 (2.84)	0.001
SAPS II		42.92 (11.84)	60.56 (12.86)	52.05 (15.19)	0.001
mNUTRIC		4.53 (1.72)	6.79 (1.52)	5.7 (1.98)	0.001

SD: standard deviation; ICU: intensive care unit; SOFA: Sequential Organ Failure Assessment; SAPS: Simplified Acute Physiology Score; mNUTRIC: modified Nutrition Risk in the Critically Ill; and intensive care unit readmission definition includes readmissions within a year. Mechanical ventilation requirements include both invasive and non-invasive modes of ventilation. The admission duration range and Nutrition Risk Screening 2002 are reported as median values, along with the 25th to 75th percentiles.

**Table 2:** Admission laboratory parameters.

Parameters (*n*, 25th–75th Percentile)	Survivors (*n* = 122)	Non-Survivors (*n* = 131)	Total (*n* = 253)	*p* Value
Complete Blood Count				
White Blood Cell (10^9^/L)	11.05 (8.7–14.5)	10.1 (6.8–16.5)	10.6 (7.8–14.7)	0.672
Lymphocyte (10^9^/L)	1.17 (0.69–1.8)	0.7 (0.4–1.26)	0.9 (0.5–1.79)	0.001
Neutrophil (10^9^/L)	7.7 (5.6–12.625)	9.2 (6–13.6)	8.7 (5.8–12.9)	0.318
Platelet (10^9^/L)	279 (194–339)	197 (124–290.5)	230 (153–318)	0.001
Liver Function Tests				
Total Bilirubin (mg/dL)	0.6 (0.4–1)	0.75 (0.5–1.4)	0.7 (0.5–1.2)	0.001
Direct Bilirubin (mg/dL)	0.2 (0.1–0.3)	0.3 (0.2–0.7)	0.2 (0.1–0.5)	0.001
Alkaline Phosphatase (IU/L)	86 (68–115)	89 (69–123)	86 (68–123)	0.705
Alanine Aminotransferase (IU/L)	18 (12–29)	20 (15–43)	19 (13–37)	0.005
Aspartate Transaminase (IU/L)	24 (17–33)	35 (23–56)	27 (20–41.5)	0.001
Gamma-Glutamyl Transferase (IU/L)	44 (30–64)	44 (22–74)	44 (26–68)	0.644
Renal Function Tests				
Creatinine (mg/dL)	0.8 (0.7–1.2)	1.1 (0.7–1.9)	0.8 (0.7–1.5)	0.001
Glomerular Filtration Rate (mL/min/1.73 m^2^)	83 (50–99)	60 (31–87)	79 (39–91)	0.001
Blood Urea Nitrogen (mg/dL)	19 (14–38)	42 (30–59)	33 (17–51)	0.001
Nutritional Status and Inflammatory Markers				
C-Reactive Protein (mg/dL)	66.5 (20.5–108.75)	101 (39–204)	85 (24–175.9)	0.001
Procalcitonin (ng/mL)	0.24 (0.08–0.87)	0.85 (0.205–8.9)	0.39 (0.12–3.18)	0.001
Albumin (g/dL)	3.22 (0.6)	2.64 (0.52)	2.92 (0.63)	0.001
Prealbumin (mg/dL)	14 (9–16)	7 (5–11.5)	10 (6–14)	0.001

**Table 3:** Regression analysis for parameters affecting first month mortality.

Predictor Parameters	Estimate	95% Confidence Interval		Standard Error	Z Score	*p* Value
		Lower	Upper			
CCI Score	0.12424	−0.11256	0.36105	0.12082	1.0283	0.304
SAPS II	0.07473	0.02528	0.12418	0.02523	2.9618	0.003
Ionotropic Support	1.11374	0.01119	2.21629	0.56253	1.9799	0.048
Mechanical Ventilation	−1.06962	−2.38678	0.24754	0.67203	−1.5916	0.111
mNUTRIC Score	0.36256	0.07218	0.65294	0.14816	2.4471	0.014
Lymphocyte Count	−0.01131	−0.4374	0.41477	0.2174	−0.052	0.958
Platelet Count	−0.00225	−0.00592	0.00141	0.00187	−1.2046	0.228
Total Bilirubin	0.57963	−0.09052	1.24977	0.34192	1.6952	0.09
Creatinine	−0.1594	−0.58614	0.26733	0.21773	−0.7321	0.464
Albumin	−1.52366	−2.3776	−0.66973	0.43569	−3.4971	0.001
Intercept	−1.13633	−5.02448	2.75182	1.98379	−0.5728	0.567

The estimate represents the log odds of mortality. CCI: Charlson Comorbidity Index; SAPS: Simplified Acute Physiology Score II; and mNUTRIC: Nutrition Risk in the Critically Ill.

**Table 4:** AUC comparison of mNUTRIC, SAPS II, mNUTRIC + SAPS II, and ROC analysis.

Scoring System	AUC	95% Confidence Interval		Standard Error		*p* Value
		Lower	Upper			
mNUTRIC	0.922	0.762	0.828	0.017		0.001
SAPS II	0.975	0.8	0.862	0.016		0.001
mNUTRIC + SAPS II	0.735	0.815	0.874	0.015		0.001
**Pairwise Delong Comparison**	**AUC Difference**	**95% Confidence Interval**		**Standard Score**	**Standard Error Difference ^1^**	***p* Value**
		**Lower**	**Upper**			
mNUTRIC—SAPS II	−0.036	−0.073	0.001	−1.951	0.18	0.051
mNUTRIC—Combined	−0.05	0.083	−0.017	−2.967	0.178	0.003
SAPS II—Combined	−0.013	−0.018	−0.009	−5.449	0.175	0.001

AUC: area under the curve; SAPS: Simplified Acute Physiology Score; and mNUTRIC: modified Nutrition Risk in the Critically Ill. The combined definition stands for a combination of the mNUTRIC and SAPS II Scores. ^1^ Given for evaluation under nonparametric assumption.

## Data Availability

The data presented in this study are available on request from the corresponding author.

## Abbreviations

The following abbreviations are used in this manuscript:

ICU	intensive care unit
MV	mechanical ventilation
NIMV	Non-Invasive Mechanical Ventilation
COPD	Chronic Obstructive Pulmonary Disease
APACHE II	Acute Physiology and Chronic Health Evaluation II
SOFA	Sequential Organ Failure Assessment
SAPS II	Simplified Acute Physiology Score II
mNUTRIC	modified Nutrition Risk in the Critically Ill
ABG	arterial blood gas
CCI	Charlson Comorbidity Index
CRP	C-reactive protein
ROC	Receiver Operating Characteristic
AUC	area under the curve
ARDS	acute respiratory distress syndrome
COVID-19	Coronavirus Disease 2019
ECMO	Extracorporeal Membrane Oxygenation
